# Pancreatic adenocarcinoma upregulated factor serves as adjuvant by activating dendritic cells through stimulation of TLR4

**DOI:** 10.18632/oncotarget.4859

**Published:** 2015-08-07

**Authors:** Tae Heung Kang, Young Seob Kim, Seokho Kim, Benjamin Yang, Je-Jung Lee, Hyun-Ju Lee, Jaemin Lee, In Duk Jung, Hee Dong Han, Seung-Hyun Lee, Sang Seok Koh, T.-C. Wu, Yeong-Min Park

**Affiliations:** ^1^ Department of Immunology, KU Open Innovation Center, School of Medicine, Konkuk University, Chungju, South Korea; ^2^ Aging Research Institute, Korea Research Institute of Bioscience & Biotechnology, Daejeon, South Korea; ^3^ Research Center for Cancer Immunotherapy, Hwasun Hospital, Chonnam National University, Hwasun, Jeollanamdo, South Korea; ^4^ Department of Microbiology, KU Open Innovation Center, School of Medicine, Konkuk University, Chungju, South Korea; ^5^ Department of Biological Sciences, Dong-A University, Busan, South Korea; ^6^ Departments of Pathology, Johns Hopkins Medical Institutions, Baltimore, Maryland, USA; ^7^ Department of Obstetrics and Gynecology, Johns Hopkins Medical Institutions, Baltimore, Maryland, USA; ^8^ Department of Molecular Microbiology and Immunology, Johns Hopkins Medical Institutions, Baltimore, Maryland, USA; ^9^ Department of Oncology, Johns Hopkins Medical Institutions, Baltimore, Maryland, USA

**Keywords:** PAUF, dendritic cells, cancer vaccines, adjuvants, TLR4

## Abstract

Dendritic cell (DC) based cancer vaccines represent a promising immunotherapeutic strategy against cancer. To enhance the modest immunogenicity of DC vaccines, various adjuvants are often incorporated. Particularly, most of the common adjuvants are derived from bacteria. In the current study, we evaluate the use of a human pancreatic cancer derived protein, pancreatic adenocarcinoma upregulated factor (PAUF), as a novel DC vaccine adjuvant. We show that PAUF can induce activation and maturation of DCs and activate NFkB by stimulating the Toll-like receptor signaling pathway. Furthermore, vaccination with PAUF treated DCs pulsed with E7 or OVA peptides leads to generation of E7 or OVA-specific CD8+ T cells and memory T cells, which correlate with long term tumor protection and antitumor effects against TC-1 and EG.7 tumors in mice. Finally, we demonstrated that PAUF mediated DC activation and immune stimulation are dependent on TLR4. Our data provides evidence supporting PAUF as a promising adjuvant for DC based therapies, which can be applied in conjunction with other cancer therapies. Most importantly, our results serve as a reference for future investigation of human based adjuvants.

## INTRODUCTION

Dendritic cell (DC) based cancer vaccines represent a promising approach [1, 2]. DCs are the most potent antigen-presenting cells, particularly in priming CD8+ T cell mediated immune responses, due to the expressions of major histocompatibility (MHC) class I and costimulatory molecules [3]. A number of preclinical and clinical studies on various cancers demonstrated that DC vaccines are safe, however, only modestly immunogenic [3, 4]. To increase the antitumor immune responses generated by DC vaccines, adjuvants should be incorporated.

The conventional adjuvants include cytokines, Toll-like receptor ligands and heat shock proteins. Cytokines such as interleukin-2 (IL-2) and granulocyte-macrophage colony-stimulating factor (GM-CSF) have been used as adjuvants in cancer vaccines [5, 6]. Most of the current cancer vaccine adjuvants are derived from bacteria, and are used to elicit innate immune responses mostly by stimulating the Toll-like receptors (TLR) [7]. Furthermore, extra-cellular and membrane heat shock proteins (HSPs) have been shown to enhance vaccine induced immune responses [8]. Numerous adjuvants have been incorporated into DC vaccines, such as Tumor necrosis factor-related activation-induced cytokine (TRANCE) and OK-432 [9, 10], a bacterial adjuvant. DC vaccines can also be used in combination with other therapies, such as using chemotherapies to modulate the tumor microenvironment, to elicit stronger antitumor immune responses [11].

Pancreatic adenocarcinoma upregulated factor (PAUF) is a secreted protein only expressed in primates [12]. It has been shown that PAUF is overexpressed in pancreatic cancers and a number of other cancers, and promotes metastasis by upregulating CXCR4 expression that may lead to increased tumor cell motility [13, 14]. Furthermore, PAUF promotes angiogenesis and vascular permeability resulting in tumor proliferation [15]. It has been reported that PAUF is an endogenous ligand for TLR2 and TLR4, and can lead to increased phosphorylation of ERK, JNK, and p-38 of the innate immunity TLR signaling pathway without activating NFkB in THP-1 cells [14]. Importantly, DCs express both TLR2 and TLR4. We therefore hypothesize that PAUF can be used as an adjuvant in DC cancer vaccine by stimulating the innate immune responses, which may lead to stronger adaptive cell mediated antitumor responses.

In the current study, we evaluated the use of PAUF as adjuvant for an antigen-specific DC vaccine in mice. We found that PAUF can induce activation and maturation in DCs, and stimulate TLR signaling pathways leading to NFkB activation. Furthermore, DCs pulsed with E7 or OVA antigenic peptide treated with PAUF can generate production of E7 or OVA-specific CD8+ T cells and memory CD8+ T cells, which correlated with long-term tumor protection against TC-1 and EG.7 tumor challenge. In addition, the antigen-specific CD8+ T cell responses induced by PAUF treated DC vaccines elicited an antitumor effect leading to prolonged survival. Finally, we've demonstrated that the mechanism of PAUF enhanced DC vaccine potency is dependent on TLR4. Taken together, our results indicate that PAUF can serve as a novel adjuvant to increase the immunogenicity of cancer vaccines.

## RESULTS

### PAUF induce maturation, activation in DCs, and activate TLR signaling pathway *in vitro*

**Figure 1:**
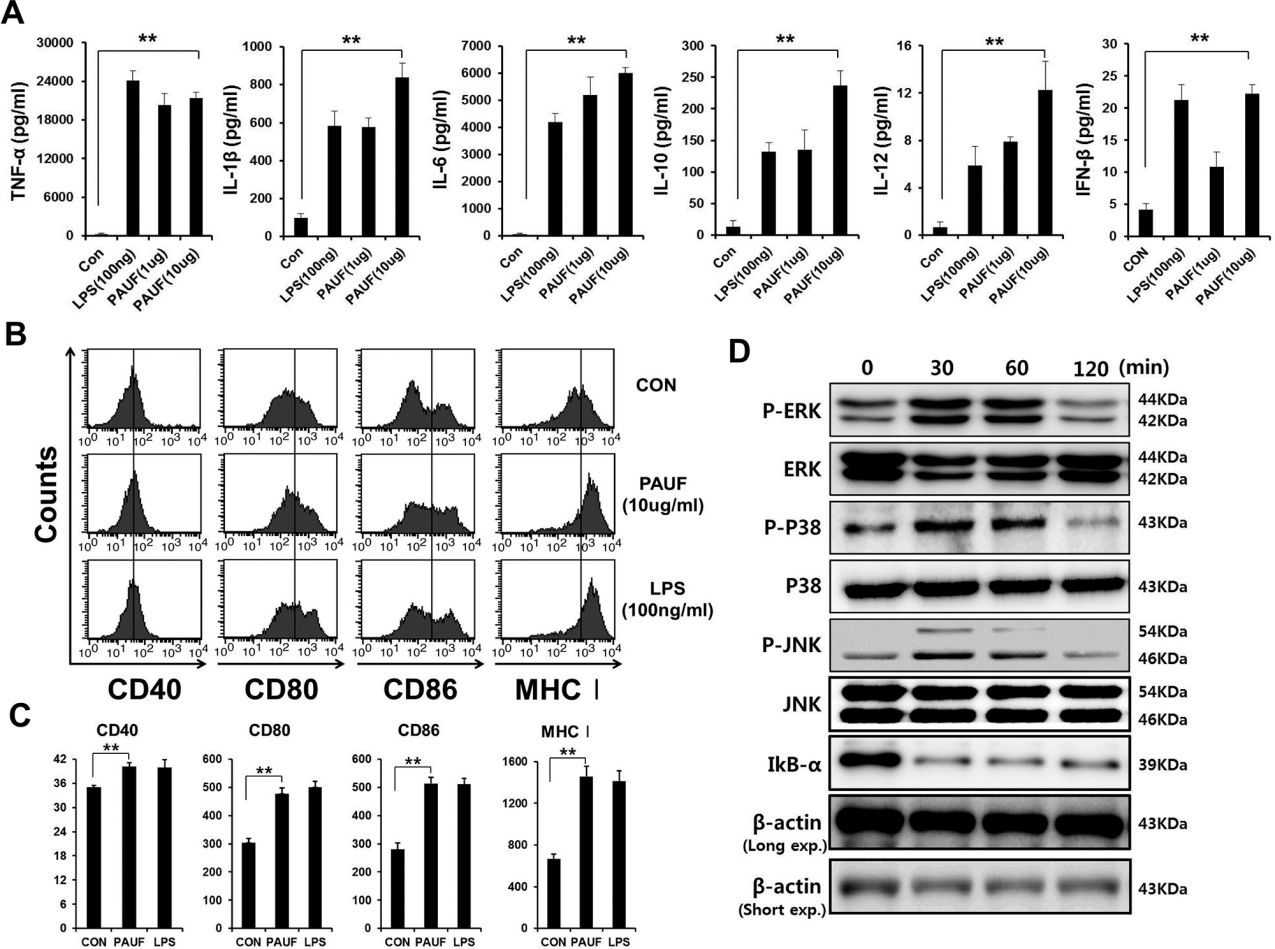
DCs maturation, activation and common TLR signal pathway activation by PAUF protein **A.** DCs activation was demonstrated by measuring pro-inflammatory cytokines and type I interferon, DCs activation factors, in culture supernatant of DCs incubated with PAUF (1 μg or 10 μg) or LPS (100 ng) as a positive control for 16 hr with ELISA. **B.** and **C.** To probe maturation level of DCs, after DCs were incubated with PAUF or LPS for 16 hr, maturation surface markers (CD40, CD80, CD86, MHC class I) were confirmed by using flow cytometry analysis after incubation with PAUF or LPS as a positive control and PBS as a negative control. **D.** To determine MAPKs and IkB-α, intracellular protein marker of activated DCs, DCs were treated with PAUF (5 μg) of time course and analyzed by using Western Blot analysis. **: *P* < 0.01.

We first determine the effects of PAUF on DCs by characterizing the pro-inflammatory cytokine expression and activation markers. As shown in Figure 1A, incubation with PAUF significantly increased the expression of pro-inflammatory cytokines TNF-α, IL-1β, IL-6, IL-10, IL-12, and IFN-β in DCs compared to untreated DCs in a dose dependent manner. Interestingly, DCs incubated with PAUF or LPS also expressed higher levels of the migration factor CCR7 (Supplementary Figure S1). In addition, DCs incubated with PAUF express higher levels of maturation surface markers CD40, CD80, CD86, and MHC class I compared to untreated DCs at a level similar to LPS treated DCs (Figures 1B–1C). These results indicate that PAUF can activate and induce maturation in DCs. PAUF have been identified to be an endogenous ligand for TLRs 2 and 4 [14]. We hypothesized that PAUF can activate the innate immunity pathway in DCs by stimulating TLRs. DCs were cultured with 5 ug of PAUF and Western Blot was used to assess various signaling proteins in the TLR signaling pathway. As shown in Figure 1D, elevated levels of P-ERK, P-P38, and P-JNK were observed in DCs after incubation with PAUF for 30 minutes up till at least 60 minutes. Furthermore, the reduction in IkB-α levels following PAUF treatment indicates NF-kB activation which results in the degradation of IkB-α. These results show that PAUF can activate innate immune responses in DCs through stimulation of TLR.

### PAUF induce maturation, activation, and migration in human DCs

**Figure 2:**
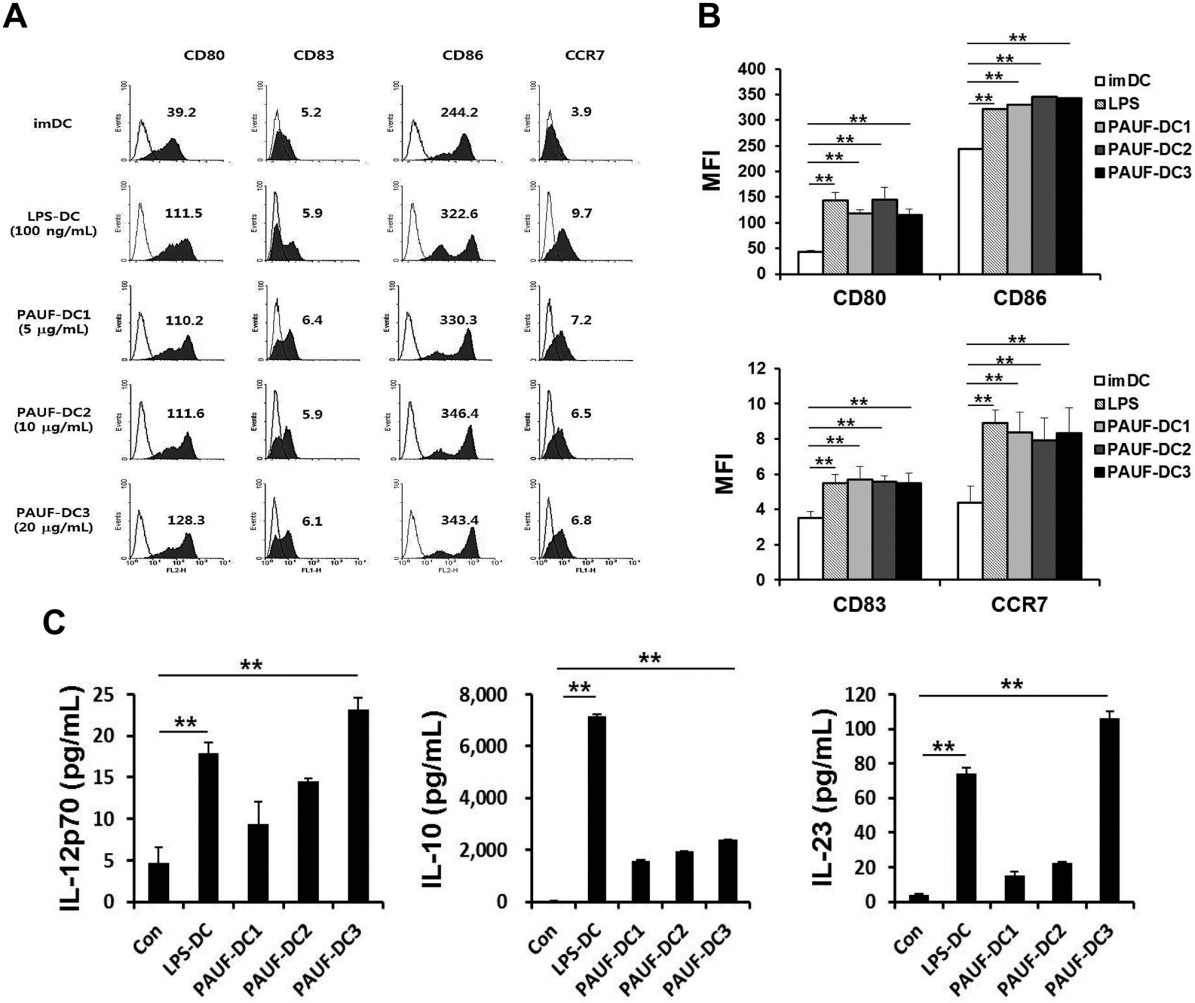
Human Dendritic cells can be activated and matured using PAUF protein **A.** To confirm maturation factor (CD80, CD83 and CD86) and migration factor (CCR7) of mature DCs compared with immature DCs (imDC), DCs (PAUF-DC1, PAUF-DC2, and PAUF-DC3) were treated with PAUF of various concentrations (5 μg, 10 μg and 20 μg/ml, respectively) or LPS (100 ng/ml; LPS-DC) for 2 days, and then analyzed by flow cytometry. Histogram shows antibody staining (in dark) relative to isotype-matched control (transparent). Data are mean fluorescence intensities (MFI) of three independent experiments. **B.** The bar graph depicts mean fluorescence intensity (MFI) of each surface markers. **C.** Cytokines (IL-12p70, IL-10 and IL-23) in the culture supernatant of PAUF (5 μg, 10 μg and 20 μg/ml) of various concentration or LPS (100 ng/ml)-treated DCs for 2 days were determined by ELISA. Immature DC culture supernatant was used as a control. **: *P* < 0.01.

Next, we characterized the effect of PAUF on human DCs. As shown in Figures 2A–2B, the expression of maturation factors CD80, CD83, and CD86 and migration factor CCR7 in human DCs were significantly increased after incubation with PAUF compared to untreated DCs (imDC) in a dose independent manner. Furthermore, PAUF (20 ug/ml) induces a higher level of cytokines IL-12p70 and IL-23, and lower level of IL-10 in human DCs compared to LPS treated human DCs (Figure 2C). These results indicate that PAUF is able to induce activation and maturation in human DCs. More importantly, the increased expression of IL-12p70 and decreased expression of IL-10 promotes polarized differentiation of naïve T cells into Th1 cells leading to enhanced activation of CTL immune responses.

### PAUF-activated DC vaccine generates antigen-specific CD8+ T cells and memory T cells *in vivo* and induces long-term tumor protection

**Figure 3:**
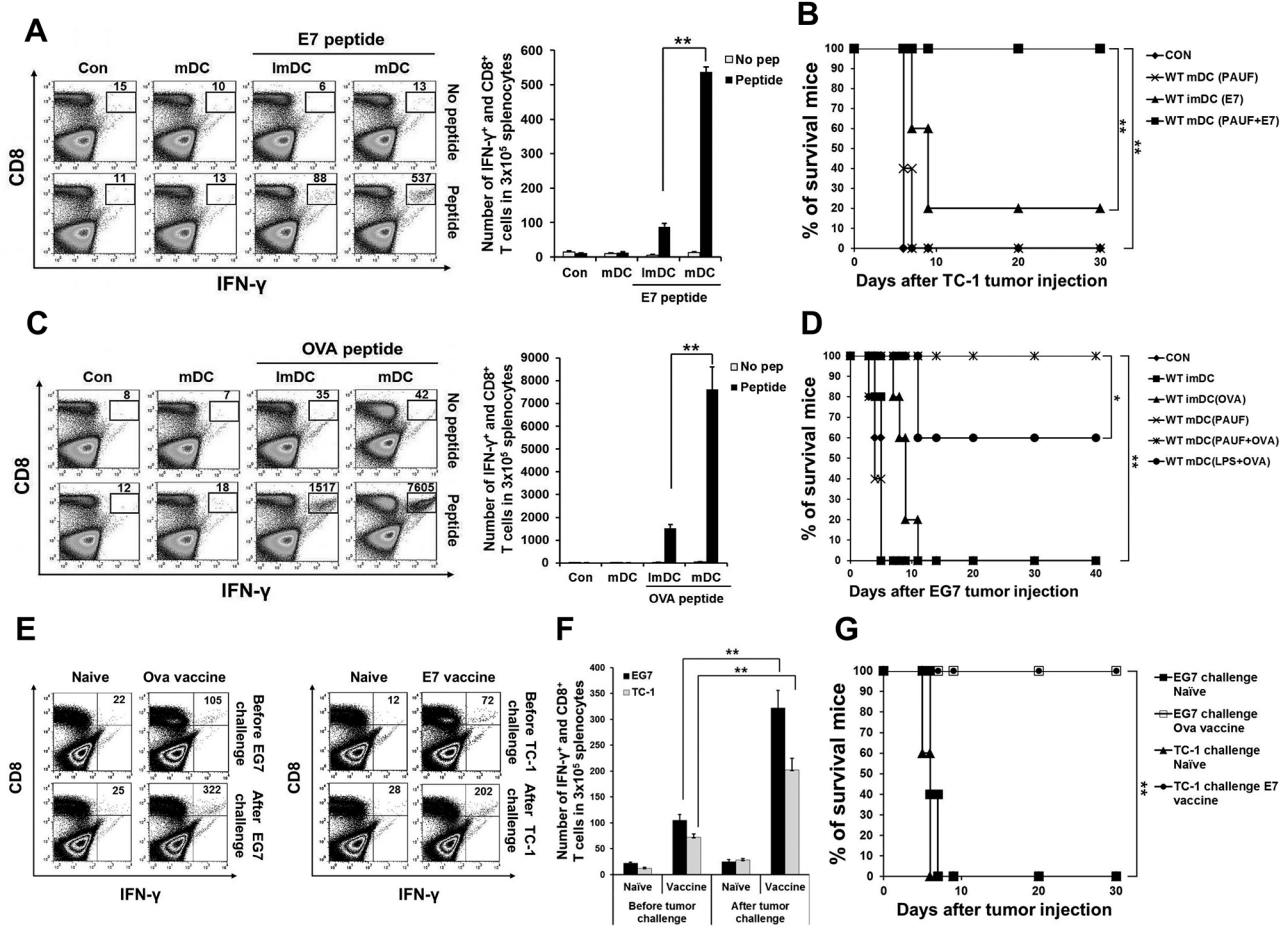
PAUF mediated DC vaccine can generate antigen specific CD8+ T cell and memory CD8+ T cell immune response and has tumor prevention effects **A.** and **C.** E7 or OVA specific CD8+ T cells were counted in splenocytes of immunized mice after various conditional DC vaccines by using flow cytometry. **B.** and **D.** Kaplan-Meier survival analysis of mice. For *in vivo* tumor prevention effects against E7 expressing TC-1 cells and OVA expressing EG.7 cells, mice were immunized with DCs and then TC-1(2.5 × 10^5^) or EG.7(5 × 10^6^) tumor cells were subcutaneously challenged after 7 days last DCs vaccination. **E.** To demonstrate *in vivo* long-term antigen (E7 and OVA) specific memory CD8+ T cells generation after PAUF mediated DC vaccine, after seven weeks of last vaccination, activated antigen specific CD8+ T cells were counted in splenocytes of only immunized mice and one week after tumor challenged mice. Naïve mice and tumor challenged mice without vaccination were used as a control. **F.** The bar graph indicated the number of antigen specific CD8+ T cells in splenocytes. **G.** Kaplan-Meier survival analysis of mice. For the test of long-term tumor protection ability after PAUF mediated DC vaccination, mice were subcutaneously challenged with TC-1(1 × 10^5^ cells/mouse) or EG.7(1 × 10^6^ cells/mouse) seven weeks after last vaccination. Same numbers of tumor cells were injected into naïve mice as a control. **: *P* < 0.01.

Next, we evaluated the potential of PAUF in generating antigen-specific adaptive cellular immune responses using PAUF activated DCs as a DC based vaccine strategy. As shown in Figures 3A and 3C, mice vaccinated with PAUF-treated DCs pulsed with antigenic peptides generated significantly higher number of activated CD8+ T cells as measured by IFNγ secretion. We then sought to investigate whether the increase in CTL activation translates into tumor protection. As shown in Figures 3B and 3D, all the mice vaccinated with PAUF treated DCs pulsed with E7 or OVA peptide stayed tumor free for at least 30 days following tumor challenge, while only one mouse from the group treated with untreated DCs pulsed with E7 peptide and three mice from the group treated with LPS-treated DCs pulsed with OVA peptide stayed tumor free for 30 days, with mice in the rest of the groups developing tumors within 10 days following tumor challenge. These results suggest that PAUF-treated DCs can activate antigen-specific CD8+ T cells capable of tumor protection. Then, we assessed whether the PAUF-treated DC vaccines can induce long-term memory. Surprisingly, stimulation by antigenic peptides still lead to generation of more activated antigen-specific CD8+ T cells in the splenocytes 7 weeks after last immunization (Figures 3E and 3F). Furthermore, a higher number of activated antigen-specific CD8+ T cells were observed after tumor challenge. In addition, the PAUF treated DC vaccines maintained their tumor protection effects 7 weeks after last vaccination in which all the vaccinated mice stayed tumor free for at least 30 days following tumor challenge (Figure 3G). These results indicate that PAUF treated DC vaccines can generate antigen-specific memory CD8+ T cells that can lead to long-term tumor prevention.

### PAUF mediated DC vaccine induces therapeutic antitumor effect and prolongs survival in mice

**Figure 4:**
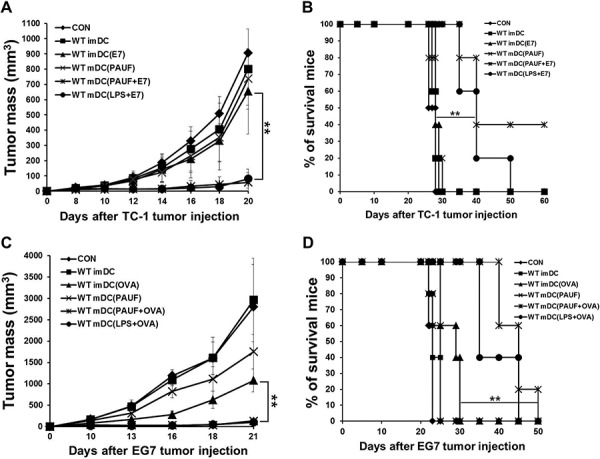
PAUF mediated DC vaccine has a significant tumor treatment effect To demonstrate tumor treatment effect, mice were subcutaneously injected with **A.** and **B.** TC-1(1 × 10^5^ cells/mouse) or **C.** and **D.** EG.7 (2 × 10^6^ cells/mouse) tumor cells. Three days after tumor cells injection, mice were treated two times at one week interval with various conditional DCs (2 × 10^6^). (A and C) Scatter plot depicting tumor growth kinetics at defined time intervals. (B and D) Kaplan-Meier survival analysis of mice. **: *P* < 0.01.

We then set out to evaluate the potential of PAUF-treated DC vaccine in clearing tumors. Interestingly, vaccination with PAUF or LPS-treated DCs pulsed with antigenic peptides suppressed tumor growth for at least 20 days in mice with 1 × 10^5 TC-1 tumor cells or 1 × 10^6 EG.7 tumor cells established for 3 days compared to other treatment regimens (Figures 4A and 4C). More importantly, 40% of mice vaccinated with PAUF treated DCs pulsed with E7 peptide survived for at least 60 days and 60% of mice vaccinated with PAUF-treated DCs pulsed with OVA peptide survived for at least 40 days while mice treated with PBS, PAUF-treated DCs only, or untreated DCs pulsed with antigenic peptides died within 30 days after tumor challenge (Figures 4B and 4D). In a more established tumor model, we challenged mice with 2 × 10^5 TC-1 tumor cells and let the tumor grow for 5 days before treatment. Consistently, as shown in Supplementary Figure S7, PAUF treated DCs pulsed with E7 peptide suppressed tumor growth for at least 20 days and significantly prolonged survival in mice compared to other vaccination regimens. These data suggest that immunization with PAUF-treated DC pulsed with antigenic peptides can induce potent therapeutic antitumor effect and prolong survival.

### PAUF mediated activation and maturation of DCs are dependent on TLR4

**Figure 5:**
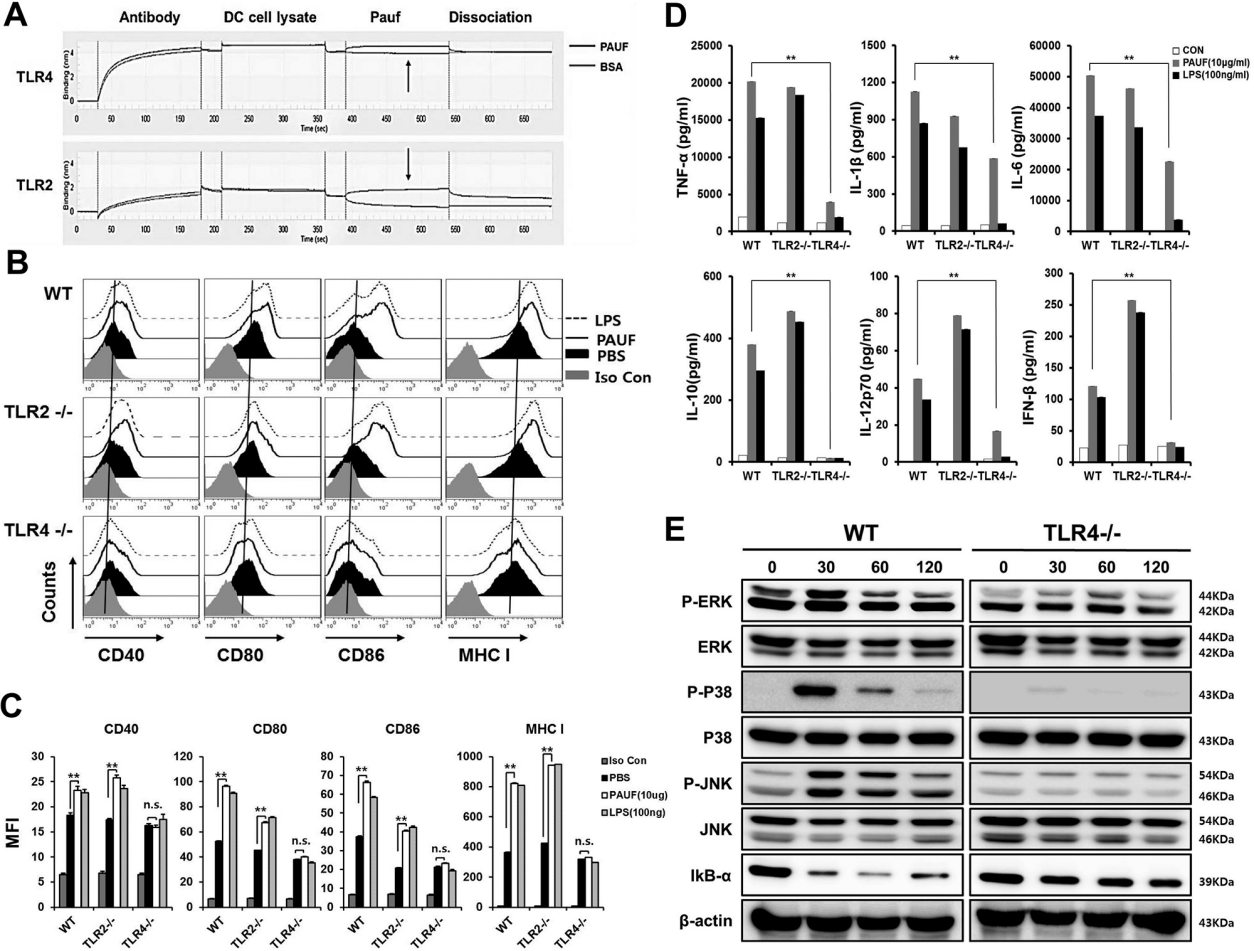
PAUF mediated DC activation and maturation depend on TLR4 **A.** To reconfirm binding of PAUF and TLR2 or TLR4 described previously, coherence between PAUF and TLR2 or TLR4 was determined with BLITZ. **B.** DCs maturation in wild type, TLR2−/− or TLR4−/− DCs after treatment with PAUF or LPS as described in Figure 1. **C.** The bar graph depicts mean fluorescence intensity of each surface marker. **D.** Bar graph depict amount of pro-inflammatory cytokines and type I interferon in wild type, TLR2−/− and TLR4−/− DCs after treatment with PAUF or LPS as described in Figure. **E.** MAPKs and IkB-α of TLR signal pathway, confirmation marker of activated-DCs, were determined by using Western Blot analysis as described in the materials and methods. **: *P* < 0.01.

PAUF has been identified as an endogenous ligand for TLRs 2 and 4. We first confirmed the affinity of PAUF protein to bind to TLRs 2 and 4 (Figure 5A). The calculated KD values between PAUF and TLR2 or TLR4 were 1.056e-8(M)(TLR2) and 1.45e-7(M)(TLR4). This data suggests that PAUF has higher binding affinity to TLR2 than TLR4. Then we wanted to determine which TLR stimulation by PAUF is responsible for activating DCs. DCs from wild type, TLR2−/−, or TLR4−/− knockout mice were incubated with or without PAUF or LPS as described above. Interestingly, increases in the expressions of various maturation markers of DCs following treatment with PAUF were abolished in DCs lacking TLR4 but not in DCs lacking TLR2 or wild type DCs (Figures 5B and 5C). Furthermore, PAUF induced increases in the expression of cytokines IL-10 and IFN-β were no longer observable, and increases in TNF-α, IL-1β, IL-6, and IL-12 expressions were reduced significantly in DCs from TLR4−/− mice (Figure 5D). Of note, the upregulation of IL-23 following treatment with either PAUF or LPS are abolished in mice lacking TLR4 but not TLR2 (Supplementary Figure S5). In addition, while P-ERK, P-P38, and P-JNK levels greatly increased with IkB-α level decreased in wild type DCs after incubating with PAUF for 30 minutes, these effects were absent in DCs lacking TLR4, but not in DCs lacking TLR2, following PAUF treatment (Figure 5E and Supplementary Figure S3), suggesting lack of NF-κB pathway stimulation. These results suggest that TLR4 is necessary for PAUF to induce activation and maturation of DCs and activate innate immune responses.

### PAUF-activated DC vaccine adjuvant effects are dependent on TLR4

**Figure 6:**
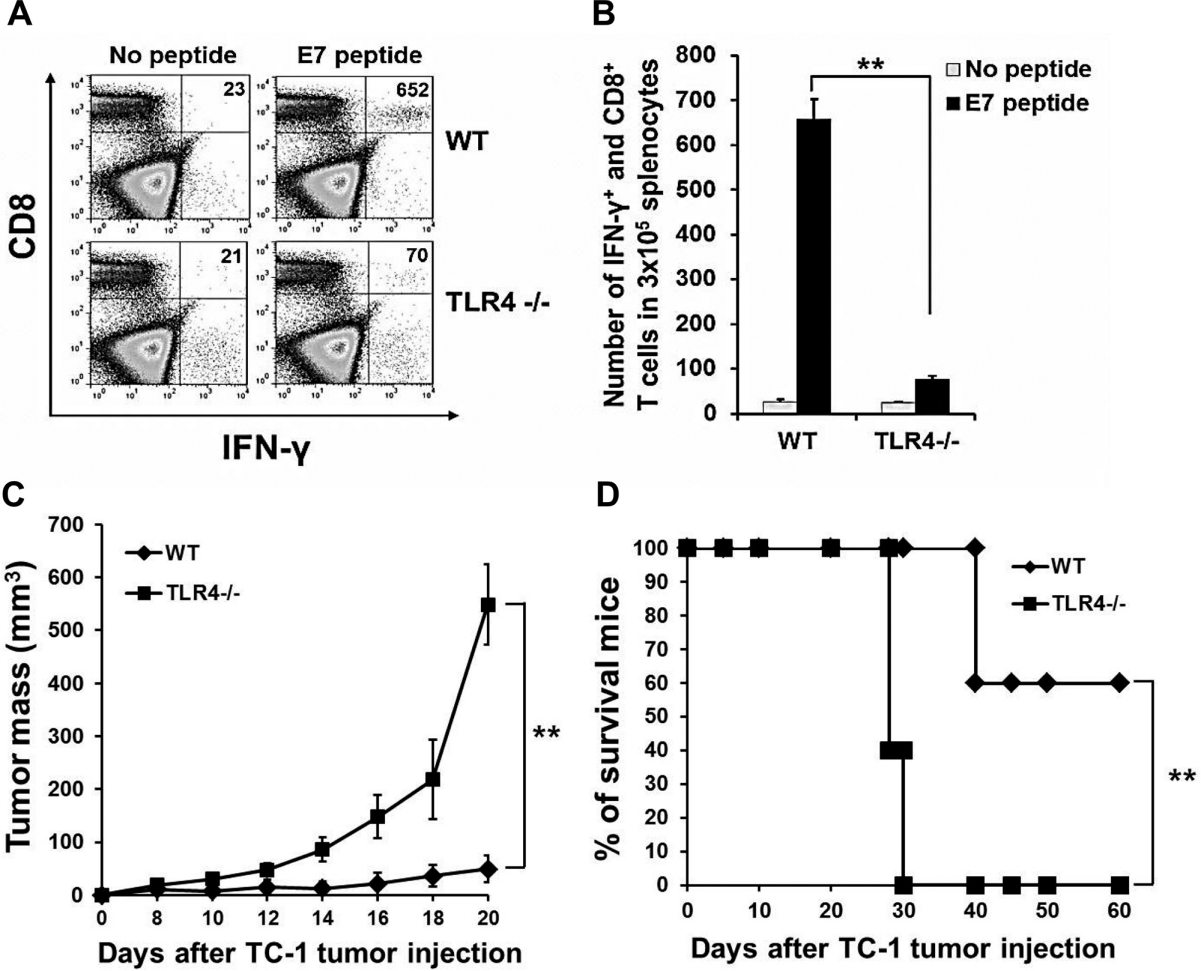
Effect of TLR4 expression on the adjuvant effect of PAUF **A.** To demonstrate TLR4 dependency of PAUF-activated matured-DCs vaccine, mice immunized with matured-Wild type or TLR4−/− DCs two times at one week interval. One week after last immunization, splenocytes were analyzed with intracellular cytokine staining and flow cytometry as described in the materials and methods. **B.** The data indicates the number of E7 specific CD8+ T cells in splenocytes of (A). **C.** and **D.** For *in vivo* TC-1 tumor treatment experiment, mice were subcutaneously injected with TC-1(1 × 10^5^) tumor cells. 3days and 10 days after tumor cells injection, mice were vaccinated with PAUF treated Wild type or TLR4−/− DCs. **: *P* < 0.01.

Finally, we further evaluated the TLR4 dependency of PAUF-activated DC vaccine. As shown in Figures 6A and 6B, while vaccination with PAUF treated wild type DCs lead to a significantly higher number of activated E7-specific T cells as measured by IFNγ secretion, vaccination with PAUF treated TLR4 deficient DCs almost completely abolished this effect. This result was also observed in the generation of OVA specific CD8+ T cells (Supplementary Figure S2). To investigate the therapeutic antitumor effects, mice were injected with TC-1 (1 × 10^5) tumor cells then vaccinated with PAUF treated wild type or TLR4−/− DCs pulsed with E7 peptide. As shown in Figure 6C, the absence of TLR4 resulted in significantly reduced tumor suppression by the PAUF DC vaccine. Furthermore, all mice vaccinated with TLR4 deficient DCs died within 30 days following tumor challenge while 60% of mice vaccinated with wild type DCs survived passed 60 days (Figure 6D). These results indicate that TLR4 is essential in PAUF-activated DC vaccine to induce antigen-specific adaptive cellular immune response and antitumor effects.

## DISCUSSION

In the current study, we evaluated the effect of PAUF in enhancing the potency of a DC based vaccine in mice. We showed that treatment with PAUF induced activation and maturation of DCs, and stimulated TLR signaling pathway resulting in activation of NFkB. Furthermore, PAUF-treated DCs pulsed with E7 or OVA peptides generated higher number of E7 or OVA-specific CD8+ T cells including memory T cells, which lead to long-term protection against TC-1 and EG.7 tumor challenge in mice. In addition, the antigen-specific CD8+ T cell immune response elicited by the PAUF-treated DC vaccine lead to potent antitumor effects and prolonged survival in tumor-bearing mice. Finally, we've demonstrated that PAUF's adjuvant effect in DC vaccines is TLR4 dependent.

To further confirm that PAUF leads to stimulation of TLR signaling pathway, we performed experiments using MyD88 knockout mice. As shown in Supplementary Figure S4, treatment with PAUF does not lead to increases in the pro-inflammatory cytokines TNF-α, IL-6, IL-10, and IL-12p70 in DCs lacking MyD88. Furthermore, expression levels of P-ERK, P-P38, and P-JNK are not elevated following PAUF treatment in DCs lacking MyD88. These results indicate that activation of DCs by PAUF is through MyD88 dependent TLR stimulation.

Although PAUF is also an endogenous ligand for TLR2, there was no significant reduction in the PAUF-induced maturation and activation of DCs in TLR2 deficient mice. These data suggest that TLR2 activation does not correlate with PAUF's immunogenicity enhancing effect in our DC vaccines. In addition, it has been reported that PAUF can bind to TLR5 and TLR6 [14]. Future studies should investigate the downstream effect of PAUF and TLR2 interaction, and PAUF's effect on TLR5 and TLR6. Previously, Park et al. reported that PAUF induced phosphorylation of ERK is inhibited when treated to mutated TLR2. This is likely due to the disruption of the binding interaction between PAUF and the mutated TLR2. Such effect is likely to be also observed in mutated TLR4 and PAUF. In order to further evaluate whether mutant molecules alter the stimulatory properties, the interactions and the precise binding sites of PAUF and TLR4 need to be characterized.

Here we show that treatment with PAUF or LPS induces production of IL-23 in maturing DCs in a TLR4 dependent manner. Interestingly, the induction of IL-23 is not observed in matured DCs co-incubated with LPS and CD40L-transfected cells (Supplementary Figure S6). It appears that the induction of IL-23 by LPS varies based on different maturation stages of DCs, while PAUF is able to induce IL-23 in DCs in various maturation stages.

In general, PAUF is a tumorigenic protein in the tumor microenvironment [16, 12]. Particularly, PAUF binds to TLR2 and TLR4 inducing an increase in expression of AP-1 regulated genes in THP-1 cells yet does not activate the NFkB pathway, resulting in promotion of escape from innate immune surveillance and tumor growth. These results are inconsistent with ours, which demonstrated immunogenic effects of TLR stimulation by PAUF leading to NFkB activation. Such discrepancy may be explained by the association of PAUF to CXCR4 in addition to TLRs in THP-1 cells, which likely inhibits TLR mediated NFkB activation. Furthermore, we show that the activation of DCs by PAUF is mainly mediated by TLR4 but not TLR2 stimulation, while in THP-1 cells the activation is mediated by TLR2. The expression levels of TLR4 and TLR2 can be very different in these two different cell lines, leading to different levels of activation of various pathways.

Some issues will have to be addressed in order for the PAUF-treated DC vaccine to be translatable. To keep the cost of producing this adjuvant reasonable, E. coli can be used to produce recombinant protein. Although PAUF possesses various pro-tumorigenic properties, here we are treating PAUF to only DCs and PAUF is washed off before DCs are injected into mice; no PAUF is actually injected into the body. Furthermore, PAUF treatment leads to activation and maturation in DCs, and is unlikely to induce oncogenic properties. Thus, we believe that the PAUF-treated DC vaccine should not carry oncogenic effects. Nevertheless the safety concern should be definitively evaluated in the future.

Until now, over 103 adjuvants have been developed and tested in vaccines [17]. The majority of these adjuvants are pathogen-associated molecular patterns (PAMPs) derived from organic or inorganic compound, or from microorganisms. Here we used PAUF, a protein of human origin, to activate DCs for the first time. The immune stimulating properties of PAUF are very similar to those of damage-associated molecular pattern molecules (DAMPs). In particular, high-mobility group box 1 (HMGB1), another ligand for TLR4 and TLR2 with human origin, also activates DCs through a TLR4 dependent manner [18]. Our result serves as a reference for further investigation of using other DAMPs such as HMGB1 to induce activation and maturation in DCs to generate antigen-specific CD8+ T cells. Although both PAMPs and DAMPs are danger signals that can activate DCs and other immune cells, their excessive presence may lead to toxicities and adverse effects, such as acute and chronic inflammation ultimately leading to cancer [19]. Future studies should evaluate whether the human origin of PAUF can help minimize the undesirable effects associated with danger signal derived adjuvants.

The current study identified PAUF as a novel human-derived adjuvant that can enhance the antigen-specific CD8+ T cell antitumor immunity of DC vaccines, by inducing activation and maturation of DCs and stimulating the innate immunity. PAUF enhanced cancer vaccines may also be used in combination with other therapies, such as using chemotherapies to reduce immunosuppression in the tumor microenvironment, to further enhance the antitumor effects. In conclusion, PAUF is a novel human based adjuvant with promising translational potential.

## MATERIALS AND METHODS

### Mice

6–8 week-old female C57BL/6 mice were purchased from Orient. C57BL/6J TLR2 knockout mice (TLR2−/−; B6.129-Tlr2tm1Kir/J) and C57BL/10 TLR4 knockout mice (TLR4−/−; C57BL/10ScNJ) at 6–8 weeks of age were purchased from the Jackson Laboratory. C57BL/6 background MyD88-deficient (MyD88−/−) mice [20] were obtained from Dr. Heung-Kyu Lee (KAIST, Daejeon, South Korea). All procedures were performed according to approved protocols and in accordance with recommendations for the proper use and care of the specific pathogen-free housing facility at Konkuk University.

### Cells

HPV-16 E7 expressing TC-1, transformed primary lung epithelial cell, and OVA expressing EG.7 (EL4 cell line transfected with the gene encoding for OVA) lymphomas were cultured in RPMI 1640 medium supplemented with 1% penicillin streptomycin and 10% fetal bovine serum.

DCs were obtained at bone marrow of wild type mouse and cultured in RPMI 1640 medium supplemented with 10% fetal bovine serum, 50 U/ml penicillin streptomycin, 2 mM L-glutamine, 1 mM sodium pyruvate, 2 mM nonessential amino acid and Granulocyte-macrophage colony-stimulating factor(GM-CSF) and grown at 37°C with 5% CO_2_.

### Reagents and antibodies

Recombinant PAUF protein was prepared as previously described [12]. Briefly, for production of PAUF protein, pcDNA3.1(+)-PAUF-Fc was constructed and transfected into the CHO/dhFr-cells. PAUF expressing clones were selected with G418 and were adapted further by the stepwise increase of methotrexate (Sigma-Aldrich, St. Louis, MO, USA). Secreted PAUF-Fc was purified using Protein A resin and then Fc was removed. The purified protein was assayed for endotoxin activity by an endpoint chromogenic LAL assay (QCL-1000; Lonza, Walkersville, MD). The endotoxin level was less than 0.1 EU/ug. Purified protein solution were kept frozen at −80°C until use. FITC-conjugated anti-mouse IFN-γ, PE-conjugated anti-mouse CD40 antibody and Mouse TNF-α, IL-1β, IL-6, IL-10, IL-12p70 ELISA Ready-SET-Go kit were purchased from eBioscience (San Diego, CA). FITC-conjugated anti-mouse CD11c antibody, PE-conjugated anti-mouse CD8a, CD80, CD86, MHC class I antibody and Mouse IFN-β ELISA kit were purchased from Biolegend (San Diego, CA). IL-23 ELISA kit was purchased from KOMA Biotech (Seoul, South Korea). BD cytofix/cytoperm Plus kit was purchased from BD Bioscience (San Jose, CA). RPMI1640, penicillin-streptomycin and fetal bovine serum (FBS) were purchased from biowest (Nuaille, France). JNK, p-JNK, P38, p-P38, ERK, p-ERK and IkB-α antibody were purchased from Cell signaling technology (Beverly, Massachusetts). β-Actin antibody was purchased from Santa cruz biotechnology (Santa Cruz, CA). LPS-EB Ultrapure was purchased from invivogen (San Diego, CA). 2-Mercaptoethanol was purchased from Gibco. Kb-restricted E749-57 (RAHYNIVTE) and Db-restricted OVA257-264 (SIINFEKL) peptides were synthesized by Anygen (Jeollanam-do, Republic of Korea). Granulocyte-macrophage colony-stimulating factor (GM-CSF) was purchased from Jw creagene (Gyeonggi do, Republic of Korea). The human DC culture medium was Iscoves modified Dulbecco's medium (IMDM) from Gibco-BRL (Grand Island, New York, USA) containing 10% FBS from PAA (Ontario, Canada). GM-CSF and IL-4 were obtained from Peprotech (Rocky Hill, New Jersey, USA). LPS was obtained from Sigma-Aldrich (St. Louis, MO, USA). Ficoll-Hypaque was purchased from Axis-SHIELD PoC AS (Lymphoprep™, Oslo, Norway). All monoclonal antibodies (mAb) against human DCs used for flow cytometry were obtained from BD Biosciences (Pharmingen, San Diego, CA, USA). Human CD14-conjugated microbeads were purchased from Miltenyi Biotec (Auburn, CA, USA).

### ELISA

DCs from wild type, TLR2−/− or TLR4−/− mouse were incubated with or without PAUF and LPS for 18 hours or time course. The culture supernatant was used for the detection of TNF-α, IL-1β, IL-6, IL-10, IL-12p70 and IFN-β by using ELISA. The production of human IL-12p70, IL-10, and IL-23 cytokines was measured over 2 days during DC maturation with PAUF or LPS.

### Flow cytometry and Intracellular cytokine staining

1 × 10^7^ splenocytes in 1ml RPMI with 10% fetal bovine serum, 1% penicilin streptomycin, 0.5% 2-mercaptoethanol were incubated for 16 hours with golgi plug, E7 or OVA peptide (1 μg/ml). Cells were washed, stained with PE-conjugated CD8a surface antibodies, fixed, permeabilized, and stained with FITC-conjugated IFN-γ antibody. Cells were analyzed on FACSCallibur using CELLQuest software. Immunophenotyping analysis of human DCs was performed by using a FACSCalibur (Becton Dickinson, San Jose, CA, USA), followed by labeling of single-cell suspensions with mAbs against human CD80-PE, CD83-FITC, CD86-PE, and CCR7-FITC or matched isotype controls (mouse IgG1 and mouse IgG2). The acquired data were analyzed with Win MDI Version 2.9 (Biology Software Net).

### Generation and maturation of human monocyte-derived DCs

Peripheral blood samples were collected from healthy donors and/or cancer patients, after obtaining informed consent according to a protocol approved by the Chonnam National University Hwasun Hospital institutional review board. Peripheral blood mononuclear cells (PBMCs) were isolated by using density gradient centrifugation with Ficoll-Hypaque, and monocytes were then isolated by positive selection with CD14-conjugated microbeads and a magnetic activated cell sorter. Monocytes of more than 95% purity were cultured at a concentration of 1 × 10^6^ cells/mL in a 6-well plate (Becton Dickinson, Franklin Lakes, New Jersey, USA) in IMDM containing 10% FBS and 1% penicillin/streptomycin supplemented with GM-CSF (50 ng/mL) and IL-4 (20 ng/mL). On day 6, the immature DCs (imDCs) were matured with LPS (100 ng/mL) or PAUF (5, 10 and 20 μg/mL), respectively for 2 days.

### Dendritic cells maturation

To confirm DCs maturation using PAUF protein, 1 × 10^7^ DCs in 1ml RPMI were incubated for 16 hours with PAUF (5 μg/ml) and LPS (100 ng/ml). Negative controls were treated with PBS alone. Cells were washed, stained with FITC-conjugated CD11c, PE-conjugated CD40, CD80, CD86 or MHC class I surface antibodies. Cells were analyzed on FACSCallibur using CELLQuest software.

### Western blot analysis

2 × 10^6^ DCs were incubated with PAUF (5 μg/ml) and LPS(100 ng/ml) for 30 min, 60 min or 120 min. Cells were scraped, washed, centrifuged, added with the protein extraction solution RIPA (50 mM Tris-Cl [pH 8.0], 150 mM NaCl, 1 mM phenylmethylsulphonyl fluoride [PMSF], 0.1% sodium dodecyl sulphate [SDS], 1% Nonidet P-40 [NP-40], and 0.5 mM EDTA; Elpis Biotech, Daejeon, Korea) and incubated for 30 min on ice. Protein concentrations were determined by Bradford protein assay kit (Pierce). Equal amount of proteins were solubilized in SDS-PAGE loading buffer (250 mM Tris-HCl, pH 6.8, 0.5 M DTT, 10% SDS, 0.5% bromophenol blue, 50% Glycerol), boiled for 10 min and then separated by SDS polyacrylamide gel eletrophoresis(SDS-PAGE) and transferred to PVDF membranes.(Traub & Co in Basel). The membranes were probed with anti-mouse JNK, p-JNK, P38, p-P38, ERK, p-ERK, IkB-α or β-actin diluted 1:1000 in 5% BSA and incubated with goat anti-mouse IgG conjugated to horseradish peroxidase (HRP) secondary antibodies. Immunoreactive bands were visualized by an enhanced chemiluminescence reaction.

### Protein binding assay using BLItz

The binding between PAUF and TLR2 or TLR4 was determined by using BLItz system (ForteBio, Menlo Park, CA) according to vendor's protocol [14, 21]. Briefly, Protein A sensors were hydrated for 10 min in PBS. Anti-Mouse CD282 (TLR2) (eBioscience) or TLR4/CD284 antibody (IMGENEX, San Diego, CA) were at 0.1 mg/mL and purified PAUF, DCs lysate and BSA were at 0.3 mg/mL. The setting was as follows : initial base line with hydrated protein A sensor for 30 sec, loading of TLR2 or TLR4 antibody for 150 sec, base line with PBS for 30 sec, loading of DC lysate for 150 sec, base line with PBS for 30 sec, association with purified PAUF or BSA for 150 sec, and dissociation with PBS for 150 sec.

BLItz system demonstrates interaction between proteins by using Bio-Layer Interferometry (BLI) technology. This system emits white light to biosensor and collects any reflected white light from biosensor. Shift of any reflected wavelengths were formed by changing the number of bound proteins to biosensor and this wavelengths were change of optical thickness to the biological layer. Therefore, shift of wavelengths was calculated to kinetic constants such as KD value. The KD of PAUF was generated by BLItz pro software analysis as a non-advanced kinetics experiment.

### Tumor treatment experiment

For TC-1 or EG.7 tumor treatment experiment, C57BL/6 mice were injected with TC-1 1 × 10^5^ cells/mouse or EG.7 1 × 10^6^ cells/mouse subcutaneously. Three days after TC-1 or EG.7 cells challenge, mice were vaccinated with PBS, untreated DCs (2 × 10^6)^, untreated DCs-pulsed E7 or OVA peptide (1 μg/ml), PAUF(5 μg/ml)-treated DCs, PAUF(5 μg/ml)-treated DCs-pulsed E7 or OVA peptide, LPS(100 ng/ml)-treated DCs-pulsed E7 or OVA peptide at footpad for a total of two times at one week intervals. Tumor growth and survival were monitored two to three times per week.

### Established tumor treatment experiment

For TC-1 tumor established treatment experiment, C57BL/6 mice were injected with TC-1 2 × 10^5^ cells/mouse subcutaneously. Five days after TC-1 cells challenge, when all of injected tumor diameter reaches to 3.5- 4 mm, mice were vaccinated with PBS, untreated DCs, untreated DCs pulsed-E7 peptide, PAUF-treated DCs, PAUF-treated DCs-pulsed E7 peptide, LPS-treated DCs-pulsed E7 peptide at footpad for a total of two times at one week intervals. Tumor growth and survival were monitored two to three times per week.

### Tumor prevention experiment

C57BL/6 mice were immunized with PBS, untreated-DCs (2 × 10^6^)-pulsed E7 or OVA peptide (1 μg/ml), PAUF(5 μg/ml)-treated DCs, PAUF treated DCs-pulsed E7 or OVA peptide for a total of two times at one week intervals. One week after last injection, mice were subcutaneously challenged with 2.5 × 10^5^ of TC-1 or 5 × 10^6^ of EG.7.

### Long-term memory T cell experiment

For the detection of long-term memory T cell, PAUF(5 μg/ml)-treated DCs pulsed with E7 or OVA peptide(1 μg/ml) immunized mice for a total of two times at one week intervals. 7 weeks after last immunization, splenocytes from vaccinated or naive mice were re-stimulated with or without E7 or OVA peptide (1 μg/ml) and Golgi plug for 16 hours. E7 or OVA specific memory T cells were measured by intracellular cellular cytokine staining method as mentioned above. For the confirm of long-term prevention effect, seven weeks after last immunization, immunized or naïve mice were subcutaneously challenged with 1 × 10^5^ of TC-1 or 1 × 10^6^ of EG.7. Tumor growth was monitored two to three times per week during 30 days. For the finding of memory T cell boosting, one week after tumor challenge, splenocytes from the mice were prepared and E7 or OVA specific T cells were measured as mentioned above.

### Statistical analysis

All data presented in this study are expressed as mean ± SD and are representative of three independent experiments performed. At least three samples per group were included in each of these experiments. Flow cytometry data and results of tumor treatment experiments were evaluated by analysis of variance (1-way ANOVA) and the Tukey-Kramer test. Individual data points were compared by Student's *t*-test. Event-time distributions for mice were compared by the Kaplan-Meier method and the logrank test. All *p* values < 0.05 were considered significant.

## SUPPLEMENTARY FIGURES



## References

[1] Davis ID, Jefford M, Parente P, Cebon J (2003). Rational approaches to human cancer immunotherapy. Journal of leukocyte biology.

[2] Banchereau J, Palucka AK (2005). Dendritic cells as therapeutic vaccines against cancer. Nature reviews Immunology.

[3] Figdor CG, de Vries IJ, Lesterhuis WJ, Melief CJ (2004). Dendritic cell immunotherapy: mapping the way. Nature medicine.

[4] Morisaki T, Matsumoto K, Onishi H, Kuroki H, Baba E, Tasaki A, Kubo M, Nakamura M, Inaba S, Yamaguchi K, Tanaka M, Katano M (2003). Dendritic cell-based combined immunotherapy with autologous tumor-pulsed dendritic cell vaccine and activated T cells for cancer patients: rationale, current progress, and perspectives. Human cell.

[5] Baek S, Kim YM, Kim SB, Kim CS, Kwon SW, Kim Y, Kim H, Lee H (2014). Therapeutic DC vaccination with IL-2 as a consolidation therapy for ovarian cancer patients: a phase I/II trial. Cellular & molecular immunology.

[6] Annels NE, Shaw VE, Gabitass RF, Billingham L, Corrie P, Eatock M, Valle J, Smith D, Wadsley J, Cunningham D, Pandha H, Neoptolemos JP, Middleton G (2014). The effects of gemcitabine and capecitabine combination chemotherapy and of low-dose adjuvant GM-CSF on the levels of myeloid-derived suppressor cells in patients with advanced pancreatic cancer. Cancer immunology, immunotherapy : CII.

[7] Urbanski HF, Sorwell KG (2012). Age-related changes in neuroendocrine rhythmic function in the rhesus macaque. Age (Dordr).

[8] Zhang X, Yu C, Zhao J, Fu L, Yi S, Liu S, Yu T, Chen W (2007). Vaccination with a DNA vaccine based on human PSCA and HSP70 adjuvant enhances the antigen-specific CD8+ T-cell response and inhibits the PSCA+ tumors growth in mice. The journal of gene medicine.

[9] Josien R, Li HL, Ingulli E, Sarma S, Wong BR, Vologodskaia M, Steinman RM, Choi Y (2000). TRANCE, a tumor necrosis factor family member, enhances the longevity and adjuvant properties of dendritic cells *in vivo*. The Journal of experimental medicine.

[10] Endo H, Saito T, Kenjo A, Hoshino M, Terashima M, Sato T, Anazawa T, Kimura T, Tsuchiya T, Irisawa A, Ohira H, Hikichi T, Takagi T, Gotoh M (2012). Phase I trial of preoperative intratumoral injection of immature dendritic cells and OK-432 for resectable pancreatic cancer patients. Journal of hepato-biliary-pancreatic sciences.

[11] Liu JY, Wu Y, Zhang XS, Yang JL, Li HL, Mao YQ, Wang Y, Cheng X, Li YQ, Xia JC, Masucci M, Zeng YX (2007). Single administration of low dose cyclophosphamide augments the antitumor effect of dendritic cell vaccine. Cancer immunology, immunotherapy : CII.

[12] Kim SA, Lee Y, Jung DE, Park KH, Park JY, Gang J, Jeon SB, Park EC, Kim YG, Lee B, Liu Q, Zeng W, Yeramilli S, Lee S, Koh SS, Song SY (2009). Pancreatic adenocarcinoma up-regulated factor (PAUF), a novel up-regulated secretory protein in pancreatic ductal adenocarcinoma. Cancer science.

[13] Lee Y, Kim SJ, Park HD, Park EH, Huang SM, Jeon SB, Kim JM, Lim DS, Koh SS (2010). PAUF functions in the metastasis of human pancreatic cancer cells and upregulates CXCR4 expression. Oncogene.

[14] Park HD, Lee Y, Oh YK, Jung JG, Park YW, Myung K, Kim KH, Koh SS, Lim DS (2011). Pancreatic adenocarcinoma upregulated factor promotes metastasis by regulating TLR/CXCR4 activation. Oncogene.

[15] Kim SJ, Lee Y, Kim NY, Hwang Y, Hwang B, Min JK, Koh SS (2013). Pancreatic adenocarcinoma upregulated factor, a novel endothelial activator, promotes angiogenesis and vascular permeability. Oncogene.

[16] Cho IR, Koh SS, Min HJ, Kim SJ, Lee Y, Park EH, Ratakorn S, Jhun BH, Oh S, Johnston RN, Chung YH (2011). Pancreatic adenocarcinoma up-regulated factor (PAUF) enhances the expression of beta-catenin, leading to a rapid proliferation of pancreatic cells. Experimental & molecular medicine.

[17] Sayers S, Ulysse G, Xiang Z, He Y (2012). Vaxjo: a web-based vaccine adjuvant database and its application for analysis of vaccine adjuvants and their uses in vaccine development. Journal of biomedicine & biotechnology.

[18] Saenz R, Futalan D, Leutenez L, Eekhout F, Fecteau JF, Sundelius S, Sundqvist S, Larsson M, Hayashi T, Minev B, Carson D, Esener S, Messmer B, Messmer D (2014). TLR4-dependent activation of dendritic cells by an HMGB1-derived peptide adjuvant. Journal of translational medicine.

[19] Rubartelli A, Lotze MT (2007). Inside, outside, upside down: damage-associated molecular-pattern molecules (DAMPs) and redox. Trends in immunology.

[20] Adachi O, Kawai T, Takeda K, Matsumoto M, Tsutsui H, Sakagami M, Nakanishi K, Akira S (1998). Targeted disruption of the MyD88 gene results in loss of IL-1- and IL-18-mediated function. Immunity.

[21] Jung ID, Shin SJ, Lee MG, Kang TH, Han HD, Lee SJ, Kim WS, Kim HM, Park WS, Kim HW, Yun CH, Lee EK, Wu TC, Park YM (2014). Enhancement of Tumor-Specific T Cell-Mediated Immunity in Dendritic Cell-Based Vaccines by Mycobacterium tuberculosis Heat Shock Protein X. J Immunol.

