# Supplementary material for: Pancreatic adenocarcinoma upregulated factor serves as adjuvant by activating dendritic cells through stimulation of TLR4
Source: Oncotarget. 2015 Aug 7;6(29):27751–62. doi: 10.18632/oncotarget.4859 (PMC4695023; doi:10.18632/oncotarget.4859)
Supplement: Supplementary file 1 [file oncotarget-06-27751-s001.pdf]

SUPPLEMENTARY FIGURES

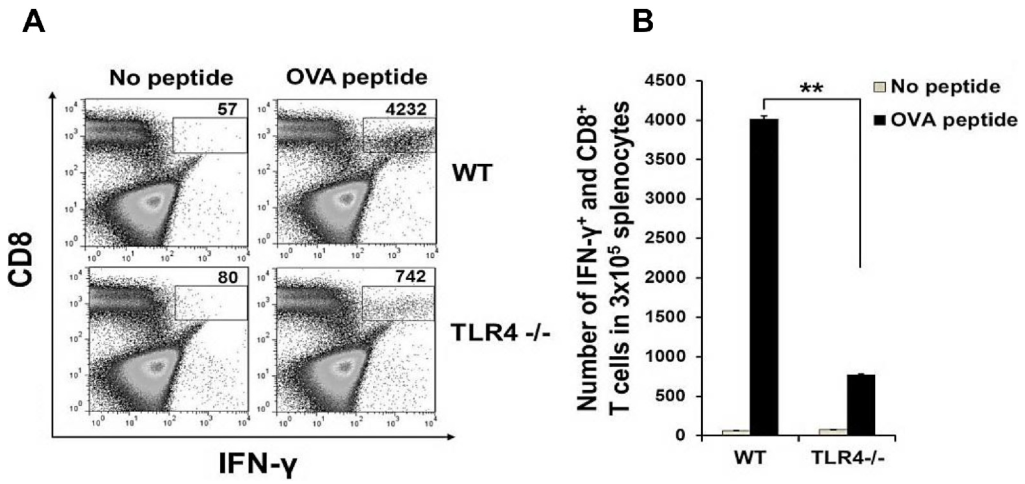

Supplementary Figure S1: OVA specific CD8+ T cells generation was highly decreased after using of PAUF treated TLR4−/− DC. A. Mice were immunized with PAUF-treated TLR4−/− or wild type DCs pulsed OVA peptide two times at one week intervals. One week after last immunization, splenocytes of immunized mice were re-stimulated with OVA peptide and determined by using intra-cellular cytokine staining as described in the Materials and Methods. **B.** The bar graph indicates the number of OVA specific CD8+ T cells in splenocytes of (A).

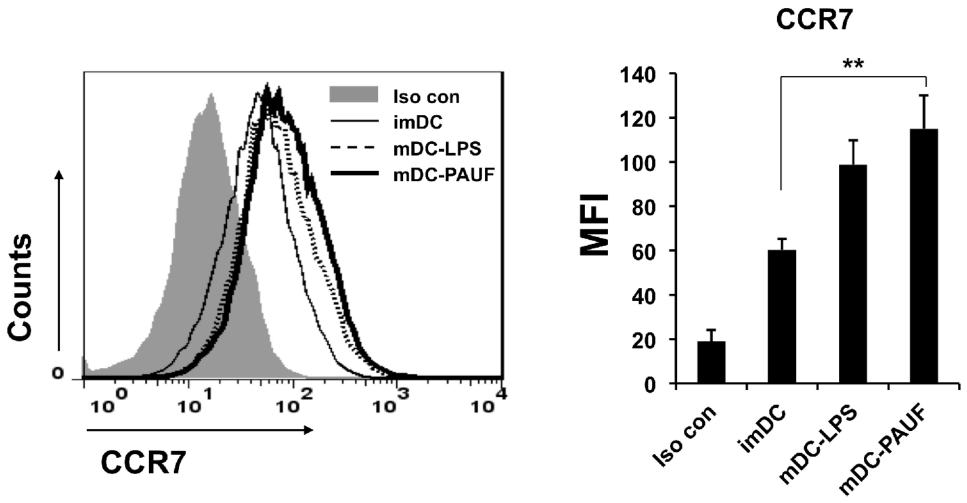

Supplementary Figure S2: CCR7 expression in PAUF treated DCs. A. DCs were treated with or without PAUF (5 µg) or LPS (100 ng) for 16 hr and stained with PE labelled mouse CCR7 antibody (eBioscience, San Diego, CA). Cells were analyzed on FACSCallibur using CELLQuest software. **B.** The data depicts mean fluorescence intensity of (A).

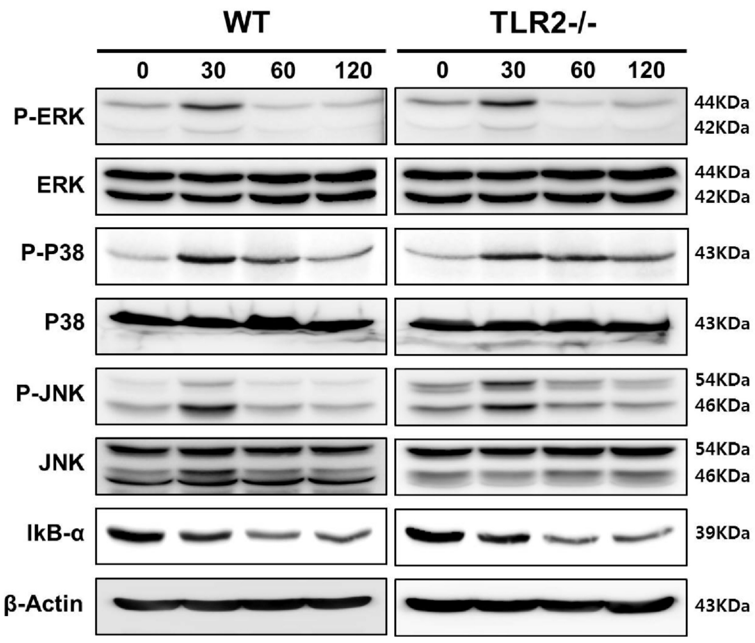

Supplementary Figure S3: PAUF mediated DC activation and maturation do not depend on TLR2. MAPKs and IkB-α of TLR signal pathway, confirmation marker of activated-DCs, were determined by using Western Blot analysis in TLR2−/− DC as described in the materials and methods.

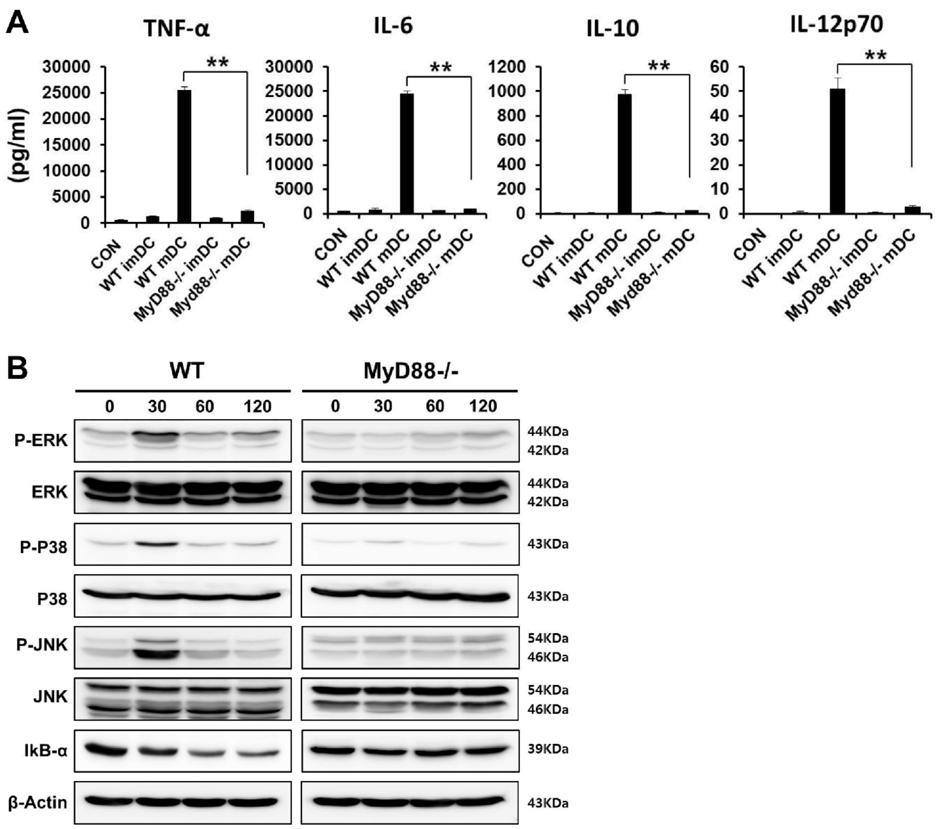

Supplementary Figure S4: PAUF mediated DC activation and maturation depend on MyD88. A. Bar graph depict amount of pro-inflammatory cytokines in wild type and MyD88−/− DCs after treatment with PAUF described in Figure. **B.** MAPKs and IkB-α of TLR signal pathway, confirmation marker of activated-DCs, were determined by using Western Blot analysis as described in the materials and methods. **:$P < 0.01$.

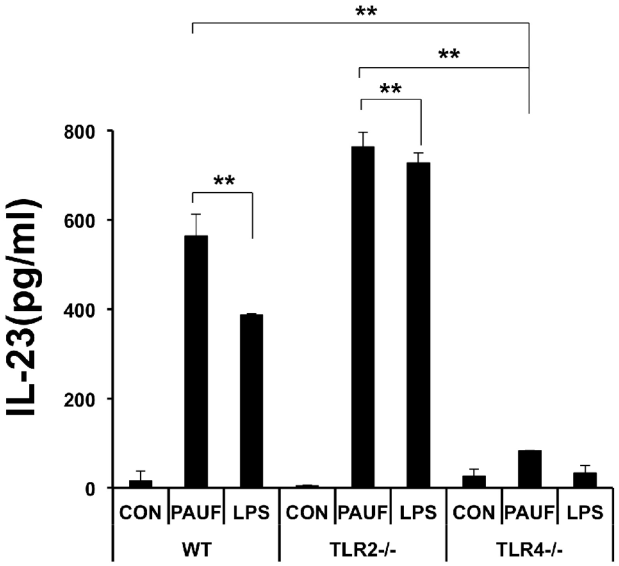

Supplementary Figure S5: IL-23 expression in mouse BMDC depend on TLR4. A. Bar graph depict amount of IL-23 in wild type, TLR4 −/− and TLR2 −/− BMDCs after treatment with PAUF and LPS described in Figure 2. **:$P < 0.01$.

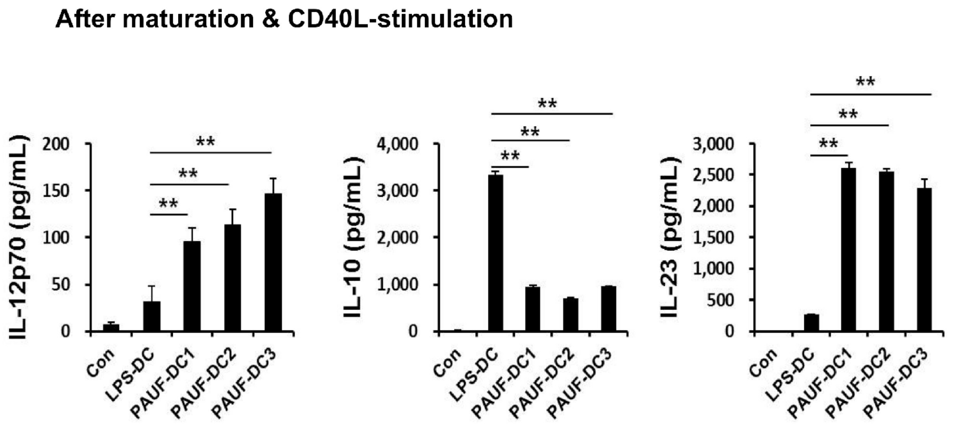

Supplementary Figure S6: Cytokine expressions in matured DCs co-incubated with PAUF and CD40L expressing cells. The production of human IL-12p70, IL-10, and IL-23 cytokines was measured after stimulation of mature DCs harvested on day 8 with CD40L-transfected J558 cells (kindly provided by Dr. P. Lane, University of Birmingham, UK) using a BD OptEIATM ELISA Set (BD Biosciences). In brief, mDCs were harvested and washed twice with PBS, followed by co-culture of mDCs (2×10^4 cells/well) with CD40L-transfected J558 cells (a gift from Dr. P. Lane, University of Birmingham, United Kingdom, 5×10^4 cells/well) in 96-well flat-bottom culture plates to mimic the interaction with CD40L-expressing Th cells, that in previous studies proved equivalent to activated CD4+ T cells and soluble CD40L [Mailliard RB, Wankowicz-Kalinska A, Cai Q et al. alpha-type-1 polarized dendritic cells: a novel immunization tool with optimized CTL-inducing activity. (17)]. After 24 h of culture, the supernatants were collected and stored at −40°C until measurement by ELISA.

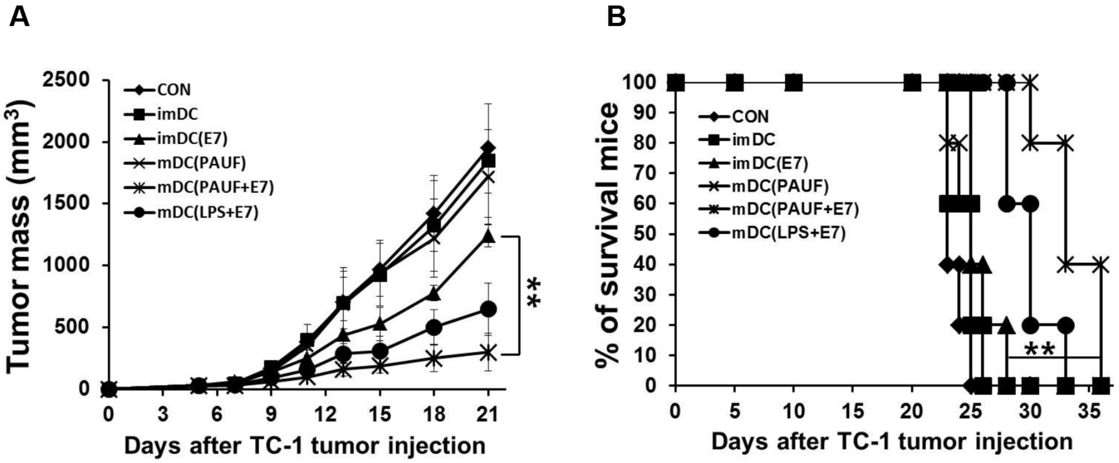

Supplementary Figure S7: PAUF activated DC vaccine has a significant tumor treatment effect. To demonstrate tumor treatment effect, mice were subcutaneously injected with **A and B.** TC-1(2×10^5 cells/mouse) tumor cells. Five days after tumor cells injection, mice were treated two times at one week interval with various conditional DCs (2×10^6). (A) Scatter plot depicting tumor growth kinetics at defined time intervals. (B) Kaplan-Meier survival analysis of mice. **:$P < 0.01$.
